# Applying Theory of Planned Behavior to Develop Family-centered Care, 2015–2016

**Published:** 2018-03

**Authors:** Forouzan ROSTAMI, Syed Tajuddin SYED HASSAN, Farideh YAGHMAEI

**Affiliations:** 1. Dept. of Nursing, Faculty of Nursing and Midwifery, Chalous Branch, Islamic Azad University, Chalous, Iran; 2. Dept. of Medicine, Nursing Unit, Faculty of Medicine and Health Sciences, Universiti Putra Malaysia, Serdang, Malaysia; 3. Dept. of Nursing, Faculty of Nursing and Midwifery, Zanjan Branch, Islamic Azad University, Zanjan, Iran

## Dear Editor-in-Chief

Family-Cantered Care (FCC), which has become the cornerstone of pediatric nursing practice, supports trust between child and family health (1). Families are recognized as an essential part of specialized childcare during illness, and they are expected to be skilled in childcare (2). The Theory of Planned Behavior (TPB) demonstrated that intention is the most important influenced by three structures: attitude, subjective norms, and perceived behavioral control (3, 4). Attitude includes performing behavior and reflects an individual’s positive or negative global evaluation of performing a particular behavior; attitude is determined by an individual’s belief about the value of the outcome of a given behavior (5). The construct known as subjective norms reflects an individual’s perceptions of social pressure to perform (or not) a behavior (4). The third construct, perceived behavioral control, refers to the degree of personal control that an individual has over the behavior (6). Hospitals in developed countries are well resourced, but many in developing countries, such as Iran, function with limited resources (7). Thus, there is a need to test FCC (based on TPB) in developing countries such as Iran.

The current study purposed to determine the extent to which pediatric nurses’ attitudes, subjective norms, and perceived behavioral control influence their intentions to provide family-centered care for child patients.

The research design of this study was experimental. Overall, 200 pediatric nurses aged 25–55 yr in two groups (100 intervention and 100 control) from hospitals affiliated to Shahid Beheshti University of Medical Sciences, Tehran, Iran (1 control and 3 intervention hospitals) in Tehran, Iran were enrolled into the study. Four hospitals were randomly selected based on the purpose of choosing hospitals that have pediatric wards for two groups (intervention and control groups). The proportionate sampling technique was employed. An educational intervention (two-day educational classes) was conducted during the first month, and its effects on the dependent variables of this study were assessed almost immediately after the intervention and three months later. In the intervention group, nurses were provided with presentations, internet-based education, booklets, and pamphlets concerning FCC. The control group received one educational pamphlet about nutrition in children. To evaluate the effect of the intervention, data were collected from both groups through researcher-made questionnaires at baseline, immediately following, and three months after the intervention.

All statistical procedures were performed using SPSS ver. 21 (Chicago, IL, USA). The level of significance was set at *P*<0.05. Descriptive and inferential statistics (two-way repeated measures ANOVA) were used to analyze the data. The mean age of participants was 36.1 yr (SD=9.1); all participants were female (100%), and none of them had specific training in FCC (93.8%). The results of repeated measure ANOVA on attitude showed that the interaction between group and test was statistically significant (F (1.42, 275) = 168.474, *P*<0.05, η^2^=0.465); therefore, the post hoc test (Bonferroni) was applied to compare the mean scores and test the related hypothesis. The results revealed a significant difference between pre- and post-test attitude scores in the intervention group (*P*<0.05).

Based on multiple regression analysis (F-statistic [F=38.006; *P*<0.01]), independent variables had a significant effect on intention. The adjusted R^2^ value increased from 0.36.3 at baseline to 0.612 after intervention. The model explains 61.2% of the variance in the intention of respondents. The value of the F-statistic (F=103.55; *P*<0.01) also revealed the significant effect of independent variables on intention. The regression model in the follow-up test was still significant according to the F-statistic (F=86.59; *P*<0.01) with an adjusted R^2^= 0.568. After 3 months, the model explained 56% of the variance in the intentions of the respondents. Three predictors (ATT, SN, and PBC) in this model explained, the most important variable, which explained 39% of the variance, followed by attitude. Two predictors (PBC and SN) in this model explained perceived behavioral control with 32% and subjective norm with 17% of the variance immediately after the intervention. The most significant predictor was ATT, followed by PBC and SN of variance in behavioral intention.

Overall, current study supports the applicability of TPB models for understanding pediatric nurses’ behavior. Attitude perceived behavioral control, and subjective norm, successively, has a significant influence on pediatric nurses’ intentions to provide family-centered care. The intervention improved the intention of these nurses to implement family-centered care in their practices. Theory-based interventions may be a potential solution to improving adherence to the behavior of healthcare professionals.
